# Elevation and seasonality modulate the leaf decomposition rates and nutrient flux of diverse species and species richness in karst river systems across China

**DOI:** 10.3389/fpls.2025.1543011

**Published:** 2025-06-04

**Authors:** Zhangting Chen, Muhammad Arif, Mengyao Tian

**Affiliations:** ^1^ School of Tourism Ecology and Environment, Guilin Tourism University, Guilin, China; ^2^ Guangxi Cultural Tourism and Wellness Integrated Development Center, Guilin Tourism University, Guilin, China

**Keywords:** single species, mixed species, leaf decomposition, river ecosystems, karst ecosystems, nutrients

## Abstract

Karst river systems (KRSs) are essential to regional biogeochemical cycling and are defined by their distinct geomorphological and hydrological features. Understanding the factors controlling litter decomposition and associated nutrient fluxes within these systems is essential for predicting ecosystem responses to environmental changes. While extensive research is underway on litter decomposition and nutrient dynamics, studies on the interactions between species richness and elevation across seasons in KRSs remain limited. This study investigates the effects of elevation (800 m, 110 m, and 60 m) and seasonality (spring and autumn) on foliage decomposition and associated nutrient fluxes in KRSs along the Li River in China. We examined the foliage decomposition of three species: *Taxodium distichum* (Linn.) Rich., *Taxodium ascendens* Brongn., and *Salix matsudana* Koidz. It included seven litter treatments in single-species and mixed-species litter bags (3 species in all single-, 2- and 3-species combinations). A total of 336 decomposition bags were used to measure leaf mass loss and nutrient release during two six-month periods at three elevations in the study area in 2023. Results revealed that seasonal changes significantly influenced initial leaf elemental concentrations, with spring samples showing the most pronounced effects. Elevation had more effect on mass loss than litter treatments, with distinct nutrient release patterns observed across different elevations. Among single species, *S. matsudana* exhibited the highest mass loss and nutrient release at lower elevations during spring, while *T. ascendens* showed the lowest rates in autumn at high elevations. Mixed-species treatments displayed different decomposition patterns, with mass loss and nutrient release following the sequence: *T. distichum × T. ascendens × S. matsudana < T. ascendens × S. matsudana < T. distichum × S. matsudana < T. distichum × T. ascendens*. Nutrient release in mixed species showed significant interactions with elevation and season, displaying both positive and negative non-additive effects. Correlation analysis indicated stronger relationships between nutrient release and mass loss in *S. matsudana* than in *T. distichum* and *T. ascendens*. This study underscores the intricate interactions between biotic and abiotic factors in KRSs. It highlights the importance of considering elevation and seasonal dynamics in ecological restoration efforts in KRSs.

## 1 Introduction

Karst landscapes cover approximately 12% of the continents, and nearly 25% of the global population depends on them for water (Chen and Arif, 2025; Ford et al., 2019). Karst terrains are highly diverse because of nutrient influxes (Green et al., 2019; Jiao et al., 2024; Liu et al., 2021). This diversity can lead to excessive algae and aquatic plant growth downstream, resulting in environmental pollution (Oleksy et al., 2020). There is an increasing awareness that karst landscapes play a contributing role in controlling biogeochemical transformations in ecosystem functioning under seasonal changes (Chen et al., 2024a; Wang et al., 2025). Factors such as seasonal variations, elevation differences, and diverse vegetation—ranging from coniferous to broad-leaved species—interact with litter decomposition and associated nutrient fluxes at different rates (Geng et al., 2024; Ma et al., 2024). These interactions significantly influence nutrient dynamics in karst river systems (KRSs), also contributing to changes in litter nutrient cycling and decomposition processes in karst regions (Yang et al., 2020, Yang et al., 2022b). Despite their critical importance, the impacts of elevation and season on litter decomposition in KRSs remain underexplored (Chen et al., 2024a). This complexity emphasizes the need for focused studies to better capture variability in litter decomposition and associated nutrient fluxes (Mora et al., 2021). While recent research has advanced our understanding of how hydrological processes influence nutrient dynamics in KRSs (Dai et al., 2021; Wang et al., 2021; Yi et al., 2021), the complex ecological processes involved in regional biogeochemical cycling are still not fully understood.

Litter decomposition plays a vital role in nutrient cycling within karst ecosystems, while surface waters in karst landscapes drive significant biogeochemical transformations (Li et al., 2021). Nutrient dynamics in the KRS can also be influenced by vegetation characteristics than other factors (Ni and Li, 2020). Aquatic vegetation, including bottom-dwelling algae and floating plants, can thrive under these conditions, affecting nutrient storage and transformation (Li et al., 2023). However, our understanding of their specific impact on the nutrient flux budget along streams remains limited. Defense mechanisms shape leaf tissue structure among vegetation types (Salgado-Luarte et al., 2023). Leaf decomposition rates are strongly influenced by their chemical composition, particularly the concentrations of nitrogen (N), phosphorus (P), lignin, and cellulose, as well as the carbon-to-nitrogen (C/N) ratio (Bertrand et al., 2006; Talbot and Treseder, 2012). Specifically, high concentrations of lignin or tannins typically inhibit decomposition, while lower C/N ratios accelerate leaf decomposition (He et al., 2019). Generally, broad-leaved trees decompose quicker than pine trees since they have a lower N concentration and a higher C/N ratio (Prescott et al., 2017). Studies indicate that elevated N concentrations promote microbial growth on leaf surfaces (Wu et al., 2023), which accelerates reproduction and enhances decomposition.

Forest ecosystem studies have shown that interactions in mixed litter decomposition change at different stages (Putra et al., 2023; Rahman et al., 2023). In the early stages, nutrients are transferred, and microbes help the process along, leading to synergistic interactions (Lyu et al., 2019). As compounds that are difficult to break down accumulate over time, antagonistic effects are likely to become more prevalent (Liu et al., 2020; Yang et al., 2022a). Translocating nutrients from high-quality to low-quality leaves enhances nutrient subsidy and relieves nutrient limitations for microorganisms through the decomposition of low-quality leaf litter (Zeng et al., 2021). For instance, this facilitates the decomposition of mixed coniferous and broad-leaved litter, demonstrating its positive impact on the ecological environment overall (Niu et al., 2020). Studies have shown that decomposition rates in the KRS are similar to those found in forest ecosystems at different elevations and seasons (Barbosa et al., 2017; Geng et al., 2024). However, when researchers used litter mixtures for decomposition, they observed non-additive but modest effects at different sampling times (Liu et al., 2020). This interaction highlights the need for comprehensive assessments of both physical (e.g., hydrological pathways, soil structure) and chemical (e.g., nutrient concentrations, biogeochemical transformations) scales to clarify the unique hydrogeological characteristics of KRSs and their complex biogeochemical processes affect regional water resources and ecological environments (Li et al., 2021).

Improved hydrologic characterization of KRSs can provide valuable insights into nutrient movement under varying conditions (Dai et al., 2021). The season and elevation affect litter decomposition and nutrient dynamics (Chen et al., 2024b). Elevation affects environmental conditions such as temperature, humidity, and vegetation types, which influence decomposition. Higher elevations usually have lower temperatures, potentially slowing down decomposition (Yang et al., 2022b). During storm events, rapid water flow at higher elevations reduces water nutrient concentrations compared to low elevation areas where nutrients accumulate due to overland flow and macropore drainage, connecting high soil nutrient levels to the stream network (Ford et al., 2019; Wang et al., 2018). Karst channels are particularly effective at retaining nutrient fluxes, even during rapid flow periods (Oehler et al., 2018; Zhao et al., 2021). This effect is largely due to temporary sediment deposits that absorb or trap nutrients, such as P (Li et al., 2021; Ni and Li, 2020). Seasonality has a pronounced effect on nutrient concentrations in karst systems, with peaks in summer and troughs during autumn and winter, driven by environmental changes (Wang et al., 2018; Yue et al., 2020; Zhao et al., 2021). In addition, climate change may accelerate phase changes in freshwater because it increases terrestrial nutrient cycling (Arif et al., 2024; Naz et al., 2024). It is predicted that ecosystem degradation can increase river transport of P, N, and suspended particles but also reduce biodiversity under these circumstances of rapid human-induced climate change (Finlay et al., 2013; Nessel et al., 2021). Recent studies indicate that these thin, fine-particle layers can impact nutrient reduction and temporary storage in stream channels (Drummond et al., 2022; Field et al., 2023), yet the role of elevation changes in streambed composition in gently sloping areas remains poorly understood. Comprehending these complex interactions is essential for fully grasping biogeochemical processes in KRSs and their implications for species and regional water resources.

KRSs in Guangxi, China, exhibit large-scale limestone landscape types, distinct hydrologic processes, and strong decomposition and nutrient transfer processes (Wang et al., 2023). It is well known for its unique landscapes and multiple ecosystem services (Liao et al., 2018; Zhang et al., 2018). The area covers approximately 2.5 million km² (Jiang et al., 2018). This is connected by surface and groundwater systems, which carry nutrients and organic matter. The alkaline nature of karst waters affects microbial activity and decomposition rates, whereas fast-flow dynamics lead to resource recycling (Wang et al., 2019). The research concluded that these systems have rapid decomposition rates and nutrient dynamics (Chen et al., 2024a). It is necessary to conduct further research in these areas to clarify the modulating factors involved in litter decomposition and nutrient flux (Wang et al., 2023). It is essential for better environmental management and protection (Li et al., 2024b). This area contains many different types of broad-leaved trees and pine habitats. Notable species attracting ecotourism include *Taxodium distichum* (Linn.) Rich., *Taxodium ascendens* Brongn., and *Salix matsudana* Koidz. Therefore, we hypothesized: (1) Elevation and seasonal variations significantly influence foliage decomposition and nutrient release in KRSs. (2) Mixed-species litter decomposes faster than single-species treatments, and certain species combinations can produce non-additive effects, influenced by both species richness and elevation. (3) The relationship between nutrient release and mass loss varies across species, with some exhibiting a stronger correlation, particularly in response to seasonal changes. This study aims to assess the effects of litter mixing, elevation, and season on litter decomposition and associated nutrient concentrations at Karts River sites in China. The main objectives are to (1) investigate how leaf species decomposition varies across elevations in KRSs. (2) Examine the season change (spring and autumn) of nutrient dynamics within single and mixed species and determine if these patterns change with elevation. (3) Explore how elevation and season change interact to influence nutrient cycling in KRSs. Such insights could aid conservation efforts by providing a more profound understanding of ecosystem dynamics in karst landscapes.

## 2 Materials and methods

### 2.1 Study area

This study encompasses three experimental stations within KRSs along the Li River in Guilin, China (**Supplementary Figure S1**). It begins in the Mao’er Mountains (25°43’38.64”N, 110°24’14.40”E) in Xing’an county. It winds its way south for 164 km, passing through Guilin (25°16’12.00”N, 110°18’00.00”E) and Yangshuo (24°46’50.88”N, 110°29’47.76”E). The subtropical monsoon weather in this region significantly influences the hydrological dynamics and seasonal flow patterns of the river (Wang et al., 2023). This results in dramatic shifts from March to September. Floods tend to occur during heavy rain, such as in 2009, when river levels rose extensively (Li et al., 2019). The Li River basin flow rate ranges from close to 300 m³ per second in the wet season to less than 50 m³ per second in the dry season (Dong et al., 2024). This decreases significantly during the dry season, but water levels fluctuate widely throughout the year. The river conditions, including water characteristics, during the incubation periods are detailed in **Supplementary Table S1**. Yangshuo experiences water level fluctuations that mirror seasonal rainfall patterns and upstream water management practices. During the wet season, water levels can soar 2–5 m higher than during dry seasons, leading to flooding of low-lying areas. In contrast, levels drop dramatically during the dry season, exposing the riverbed. Water levels fluctuate from 1.5 m in the dry season to 6 m at peak flood levels, impacting navigation and other ecosystems. This contributes to the distinct ecological and hydrological features of the river. For example, it flows between 800 and 60 m above sea level (ASL) from the Mao’er Mountains to Yangshuo. The dramatic limestone peaks in this karst landscape create unique microclimates that influence local vegetation and soil types.

Human influences such as ecotourism activities and subtropical monsoon conditions also affect this ecosystem. These conditions are characterized by high humidity and significant annual rainfall (Zhang et al., 2018). The Li River basin boasts a diverse range of ecosystems, from subtropical forests to riparian plant communities. Its boundaries are home to bamboo, camphor trees, various shrubs, grasses, and other species (Chen et al., 2024a). Soil types and hydrological conditions are closely linked to vegetation patterns. Riverine vegetation along riverbanks plays a crucial role in maintaining ecological equilibrium, providing habitat for wildlife, and stabilizing riverbanks. However, deforestation, land conversion to agriculture, and tourism contribute to habitat loss and reduced biodiversity (Li et al., 2024b). Conservation efforts focus on protecting native vegetation, restoring degraded habitats through reforestation, and employing sustainable land management practices for maximum ecological benefit. In this study, flooding-tolerant tree species planted along the Li River were selected, including *Taxodium distichum* (Linn.) Rich., *Taxodium ascendens* Brongn., and *Salix matsudana* Koidz. These coniferous and broad-leaved tree species can survive in the wet season and germinate after the water level recedes but also increase the landscape value of the riverbank and enhance the beauty of the place.

### 2.2 Experimental design and sampling

In late March (spring) and early September (autumn) of 2023, *T. distichum*, *T. ascendens*, and *S. matsudana* plants with similar growth characteristics were selected from the riparian zone at three elevations of the Li River basin. Only well-developed, mature branches free of pests and diseases were chosen. Healthy, fresh leaves were collected from branches at different heights and orientations. Leaves from the same tree species were then thoroughly mixed and placed in plastic bags, which were subsequently numbered. After collection, a portable electronic balance was utilized to accurately measure 15.00 g of fresh leaves from the same tree species, as a single-species group, including two pines (*T. distichum* and *T. ascendens*) and one broadleaf species (*S. matsudana*). In pairwise mixed combinations, each species weighs 7.50 g in equal proportions mixed. This includes three foliage treatments: *T. distichum × T. ascendens, T. distichum × S. matsudana, and T. ascendens × S. matsudana*. When the three species are combined as *T. distichum × T. ascendens × S. matsudana*, each species weighs 5 grams. We packed each type of sample in the size of 20 cm × 20 cm, and the mesh size was 0.65 mm litter bags. This study implemented seven foliage treatments, with 168 samples collected at each sampling time, resulting in a total of 336 samples (7 litter treatments × 8 samples × 3 elevations × 2 seasons). This allowed for six-month decomposition periods under seven different foliage treatments. The study was based on naturally controlled experiments, seasonal changes (late March–spring and early September–autumn), and random flooding times at heights between 800 m ASL in the Mao’er Mountains (site 1), 110 m ASL in Guilin City (site 2), and 60 m ASL in Yangshuo (site 3).

Decomposition bags were placed on March 29, 2023, and September 2, 2023, respectively, as part of ecosystem restoration efforts. We cleared any debris from the sediment surface prior to placement. Moreover, we secured the decomposition bags with plastic nets, bamboo sticks, and wire to prevent animal damage and water-induced erosion (Liu et al., 2019). In addition, we collected 5 samples for each litter treatment and packed them into the marked envelope for the determination of initial leaf dry weight and leaf traits. We then transferred these bags back to the laboratory, labeled them, and dried them until they reached an even weight distribution. Before crushing and sieving, we recorded their initial dry weights to determine their elemental concentration.

After the 180-day decomposition duration, we stored the samples in an icebox and returned them to the laboratory for further processing. We carefully examined the sediment and debris attached to the decomposition bag, as well as the plant roots and benthic animals entering it. We then stored some samples in a -80°C refrigerator for further analysis (Slade and Riutta, 2012). Furthermore, we cleaned the decomposition residue with pure water and then dried it in an oven at 60°C to a constant weight. Samples collected at both intervals were subjected to two distinct seasonal treatments, including natural flooding and three elevational environments. We kept any changes to environmental variables natural; these were not considered within the scope of this study.

### 2.3 Laboratory analyses

The remaining dry mass of the sample was accurately weighed using a balance with a precision of 1/10,000 (Shanghai Shunyu Hengping Scientific Instrument Co., Ltd., FA2004) (Feijó Delgado et al., 2013). To determine the nutrient concentration in the leaves, the sample was finely ground with a ball mill (Leech MM400, Ball Mill, Germany) and sifted through a 65-mesh (0.3-mm) sieve. The sample was then dried again and weighed according to measurement index requirements (Fletcher et al., 2021). C and N concentrations were analyzed using a German ZNS-O element analyzer (ZNS-O-Vario EL cube; Heraeus Elementar, Hanau, Germany). Total P concentration was digested using a microwave digester (SpeedWave WS-4) and subsequently measured with an inductively coupled plasma spectrometer (ICP-AES).

### 2.4 Calculation methods for leaf-dry mass loss and nutrient release during mixed leaf species decomposition

The leaf dry mass loss (%) and nutrient release (%) were calculated using the following methods (Liu et al., 2017; Vogado et al., 2020; Yang et al., 2020):


$$\text{ML}(\%)=({\text{M}}_{0}-{\text{M}}_{\text{t}})/{\text{M}}_{0}\times 100\%$$

$$\text{NL}(\%)=({\text{M}}_{0}{\text{C}}_{0}-{\text{M}}_{\text{t}}{\text{C}}_{\text{t}})/{\text{M}}_{0}{\text{C}}_{0}\times 100\%$$

ML is dry mass loss (%). NL is the nutrient release (%). M_0_ refers to the initial dry weight of the sample (g), C_0_ is its initial nutrient concentration of the sample (g·kg^-1^), M_t_ is the dry weight of the sample after decomposition time (g), and C_t_ is the nutrient concentration of the sample at decomposition time (g·kg^-1^) (Liu et al., 2017; Smart et al., 2017).


$${\text{ML}}_{\text{e}}=\frac{\Sigma_{i}ML_{\dot{i}}\times M_{i}}{\Sigma_{i}M_{i}}\times 100\%$$

$$\text{Mixing effect }\Delta =\frac{ML_{\dot{0}}\times ML_{e}}{ML_{e}}\times 100\%$$

ML_e_ is the expected mass loss of mixed species, and ML_i_ is the mass loss of species i. M_i_ is the weight of mixed species i; ML_0_ is the actual mass loss of mixed species. At the same time, the expected mass loss of mixed leaves was calculated according to the average mass loss of individual species, and the mixing effect of leaf decomposition was calculated combined with its actual mass loss to explore whether the mass loss of mixed decomposition could be predicted by its component species. Non-additive effects in mixed leaf litter decomposition exist if the mixed decomposition rate and nutrient release are different from the expected results based on the decomposition rate of each individual leaf species. This effect can be manifested as a synergistic effect, that is, mixed decomposition is faster than single decomposition (p<0.05). It can also be manifested as an antagonistic effect, that is, mixed decomposition is slower than expected (Zhang et al., 2016).

### 2.5 Statistical analyses

To assess differences in initial element concentration and ratio across different litter treatments and elevations for each sample batch, we used an independent sample T-test. A one-way ANOVA was performed to analyze variations in initial element concentration and ratio at the litter treatment level between seasonal sample batches at different elevations (Pan et al., 2013). A two-way ANOVA was conducted to examine the effects of litter treatment, elevation, and their interactions on dry mass loss and nutrient release across seasonal periods (Wang et al., 2018). Additionally, a one-way ANOVA was used to determine whether mass loss and nutrient release in mixed-species treatments significantly differed from expected values. A mixed effect greater than 0 indicates a promoting effect, or a positive additive effect (p < 0.05), whereas a mixed effect less than 0 suggests an antagonistic effect (Zhang et al., 2016). Furthermore, a Pearson correlation test was conducted to analyze the relationship between nutrient release and mass loss of pine and broadleaf species in river ecosystems (Zheng et al., 2024). All data analyses were performed using SPSS 22.0 (IBM, Chicago, USA) and Origin Pro 2023 (OriginLab Corp., USA).

## 3 Results

### 3.1 Seasonal changes affect the initial element concentration of single and mixed litter treatments at different elevations

The initial element concentrations and their ratios varied significantly across litter treatments at all three elevations (**Table 1**). Notably, the standard error variance was relatively lower at the lower elevation (60 m) compared to the upper elevations (800 m) and middle elevations (110 m). Single-type foliage exhibited higher initial C and C/N ratios than mixtures. Conversely, mixed-type foliage demonstrated higher initial TN and N/P ratios compared to single-type foliage.

**Table 1:** The initial element concentration and ratio (mean ± S.E.) of each litter treatment at different altitudes in river ecosystems.

Litter treatment	Elevation (m)	TC (mg·g^-1^)	TN (mg·g^-1^)	TP (mg·g^-1^)	C/N	C/P	N/P
Td	800	478.64 ± 1.74c	16.30 ± 0.21c	1.85 ± 0.09c	29.39 ± 0.42a	261.78 ± 13.51a	8.90 ± 0.36a
	110	507.58 ± 1.29b	20.80 ± 0.92a	2.24 ± 0.08b	24.57 ± 0.97b	227.95 ± 8.05b	9.40 ± 0.76a
	60	519.42 ± 0.90a	18.14 ± 0.28b	2.57 ± 0.04a	28.66 ± 0.43a	202.40 ± 3.26b	7.07 ± 0.21b
Ta	800	499.86 ± 1.87a	14.22 ± 0.49c	2.01 ± 0.13a	35.31 ± 1.14a	254.01 ± 17.72a	7.19 ± 0.40a
	110	484.46 ± 0.78c	15.32 ± 0.31b	1.95 ± 0.05a	31.68 ± 0.68b	248.81 ± 6.51a	7.87 ± 0.27a
	60	494.50 ± 1.02b	18.54 ± 0.21a	2.01 ± 0.37a	26.69 ± 0.31c	203.77 ± 14.15b	7.63 ± 0.51a
Sm	800	447.96 ± 1.31a	31.20 ± 0.26a	1.76 ± 0.03b	14.36 ± 0.11a	254.31 ± 4.44a	17.71 ± 0.34a
	110	447.14 ± 1.34a	30.88 ± 0.32a	2.13 ± 0.09a	14.49 ± 0.13a	211.21 ± 9.37b	14.59 ± 0.71b
	60	446.66 ± 1.01a	30.02 ± 0.57a	2.00 ± 0.05a	14.90 ± 0.26a	224.32 ± 5.1b	15.08 ± 0.44b
Td × Ta	800	498.69 ± 2.56b	17.35 ± 0.38b	2.02 ± 0.09b	28.79 ± 0.57a	248.54 ± 9.52a	8.64 ± 0.32a
	110	493.31 ± 3.01b	18.33 ± 0.3ab	2.26 ± 0.05a	26.94 ± 0.57b	218.3 ± 4.61b	8.11 ± 0.17a
	60	507.91± 0.33a	18.72 ± 0.27a	2.31 ± 0.05a	27.15 ± 0.39b	220.45 ± 4.86b	8.13 ± 0.29a
Td × Sm	800	465.4 ± 1.24c	23.9 ± 0.54b	1.86 ± 0.04b	19.51 ± 0.46a	250.63 ± 5.42a	12.86 ± 0.3a
	110	474.4 ± 2.02b	25.18 ± 0.18a	1.98 ± 0.08b	18.84 ± 0.1a	241.31 ± 9.76a	12.81 ± 0.56a
	60	480.4 ± 0.51a	24.74 ± 0.17ab	2.2 ± 0.02a	19.42 ± 0.15a	218.72 ± 1.75b	11.26 ± 0.13b
Ta × Sm	800	473.06 ± 4.16a	26.31± 0.74a	1.87 ± 0.04b	18.03 ± 0.46a	253 ± 7.14a	14.07 ± 0.5a
	110	461.44 ± 2.54b	25.99 ± 0.42a	1.98 ± 0.09b	17.77 ± 0.25a	235.51 ± 11.23ab	13.24 ± 0.54a
	60	465.8 ± 1.02ab	25.76 ± 0.49a	2.2 ± 0.05a	18.11 ± 0.38a	212 ± 4.91b	11.73 ± 0.42b
Td × Ta × Sm	800	476.03 ± 2.23b	25.94 ± 0.33a	2.1 ± 0.05a	18.37 ± 0.31c	226.97 ± 5.84a	12.36 ± 0.27a
	110	481.43 ± 1.71ab	24.89 ± 0.45a	2.01 ± 0.03a	19.37 ± 0.35b	240.1 ± 3.46a	12.42 ± 0.35a
	60	483.79 ± 1.54a	23.71 ± 0.25b	2.07 ± 0.04a	20.41 ± 0.19a	233.63 ± 4.6a	11.45 ± 0.23b

Means with different lowercase letters represent significant differences at p < 0.05 according to the independent sample t-test. The abbreviations in the table are *Taxodium distichum* (Td), *Taxodium ascendens* (Ta), *Salix matsudana* (Sm), total (T), carbon (C), nitrogen (N), and phosphorus (P). Sample size was n = 336.

The interactions among litter treatments, elevation, and the combination of litter treatments and elevation significantly influenced the initial element content of all leaf types in both spring and autumn. However, there were some exceptions: in spring, litter treatments did not significantly affect C/N ratio, and in autumn, elevation did not substantially influence C, N concentrations, C/N, and N/P ratios, nor did the interaction between leaf types and elevation contribute to C/P ratios (**Table 2**). The effects were remarkably pronounced in spring, as samples collected during this season exhibited more substantial responses in terms of litter decomposition and nutrient release patterns. Additionally, litter treatments were more influential when comparing elevation factors.

**Table 2:** Effect of litter treatments and elevation on the initial element concentrations and ratios of leaf species during different seasons in river ecosystems.

Index	Factor type	F value	
		Spring	Autumn
Carbon (C) concentration (mg·g^-1^)	Litter treatment	336.371***	73.982***
	Elevation	39.415***	2.334
	Litter treatment × Elevation	23.546***	5.687***
Nitrogen (N) concentration (mg·g^-1^)	Litter treatment	462.321***	82.968***
	Elevation	8.127**	1.118
	Litter treatment × Elevation	11.110***	7.693***
Phosphorus (P) concentration (mg·g^-1^)	Litter treatment	3.113*	28.529***
	Elevation	12.214***	8.113*
	Litter treatment × Elevation	2.216*	2.457*
C/N ratio	Litter treatment	479.918***	122.657***
	Elevation	18.217***	0.967
	Litter treatment × Elevation	17.368***	14.784***
C/P ratio	Litter treatment	0.429	38.981***
	Elevation	28.428***	6.314*
	Litter treatment × Elevation	3.229**	1.312
N/P ratio	Litter treatment	161.362***	36.985***
	Elevation	19.528***	4.718
	Litter treatment × Elevation	4.213***	4.319**

According to the two-way ANOVA, the statistical differences are significant at p < 0.001***, P < 0.01**, and P < 0.05*. Sample size was n = 336.

The mass loss of all samples was notably affected by various litter treatments and elevations (**Table 3**). Elevation exerted a more pronounced influence compared to litter treatment. Furthermore, this effect was significantly more pronounced during spring, as indicated by higher effect values. In addition, when considering litter treatment and elevation together in both seasons, statistical tests did not yield significant results.

**Table 3:** Effect of litter treatments and elevation on mass-loss rates during different seasons in river ecosystems.

Index	Factor type	F value	
		Spring	Autumn
Mass loss (%)	Litter treatments	32.312***	16.867***
	Elevation	34.986***	19.793***
	Litter treatments × Elevation	0.331	1.124

According to the two-way ANOVA, the *** denotes statistically significant differences at p < 0.001. Sample size was n = 336.

The release rates of C, N, and P in all samples were notably influenced by different litter treatments (**Table 4**). The patterns mirrored those of elevation factors, except for the N release rate in spring, which did not show significance. Likewise, the joint effect of litter treatments and elevation on these nutrient concentrations was largely significant, apart from C and N release in autumn.

**Table 4:** Effect of litter treatments and elevation on nutrient release from leaves during different seasons in river ecosystems.

Index	Factor type	F value	
		Spring	Autumn
Carbon release rate (%)	Litter treatments	6.127***	16.416***
	Elevation	47.213***	45.129***
	Litter treatments × Elevation	5.301**	2.018
Nitrogen release rate (%)	Litter treatments	20.224***	39.108***
	Elevation	2.332	30.238***
	Litter treatments × Elevation	3.225*	2.027
Phosphorus release rate (%)	Litter treatments	42.416***	11.109***
	Elevation	64.968***	7.692**
	Litter treatments × Elevation	10.318***	3.227*

According to the two-way ANOVA, the statistical differences are significant at p < 0.001***, P < 0.01**, and P < 0.05*. Sample size was n = 336.

**Figure 1:**
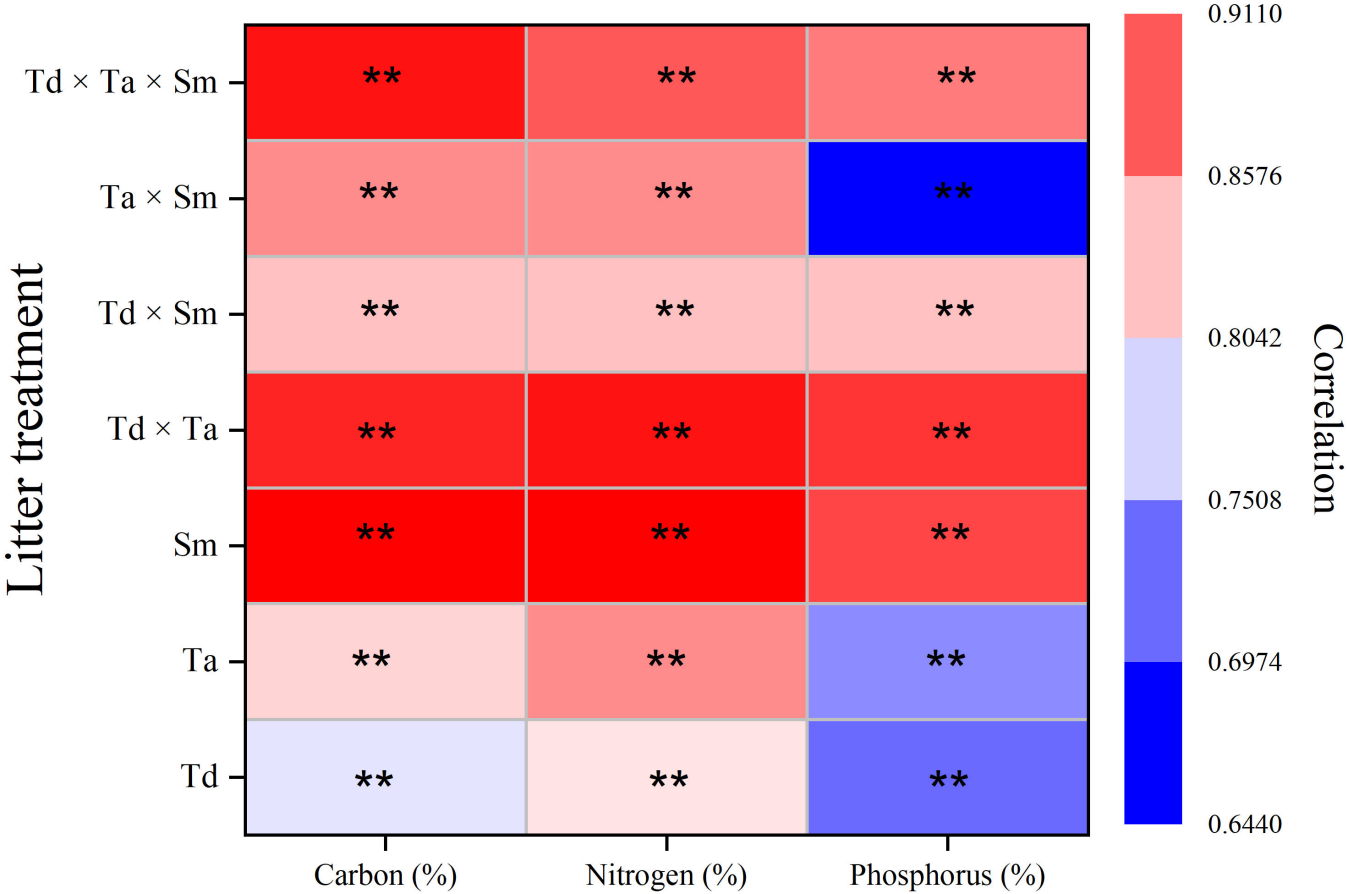
Heat map of Pearson’s correlation of leaf species nutrient release and mass loss of litter treatments in river ecosystems. **The correlation is significant at the 0.01 level (two-tailed). Abbreviations in the figure are *Taxodium distichum* (Td), *Taxodium ascendens* (Ta), and *Salix matsudana* (Sm). Sample size was n = 336.

The release rates of C, N, and P from all litter treatments exhibit significant correlations with their rates of dry mass loss (**Figure 1**). Particularly, the correlation strengths for C and N were notably stronger in comparison to P. Furthermore, this correlation is more pronounced in broad-leaf tree species as opposed to pine trees. Additionally, these correlations are more prominent in mixed-species environments compared to monocultures.

### 3.2 Mass loss of single and mixed litter treatment at different elevations under seasonal changes

**Figure 2:**
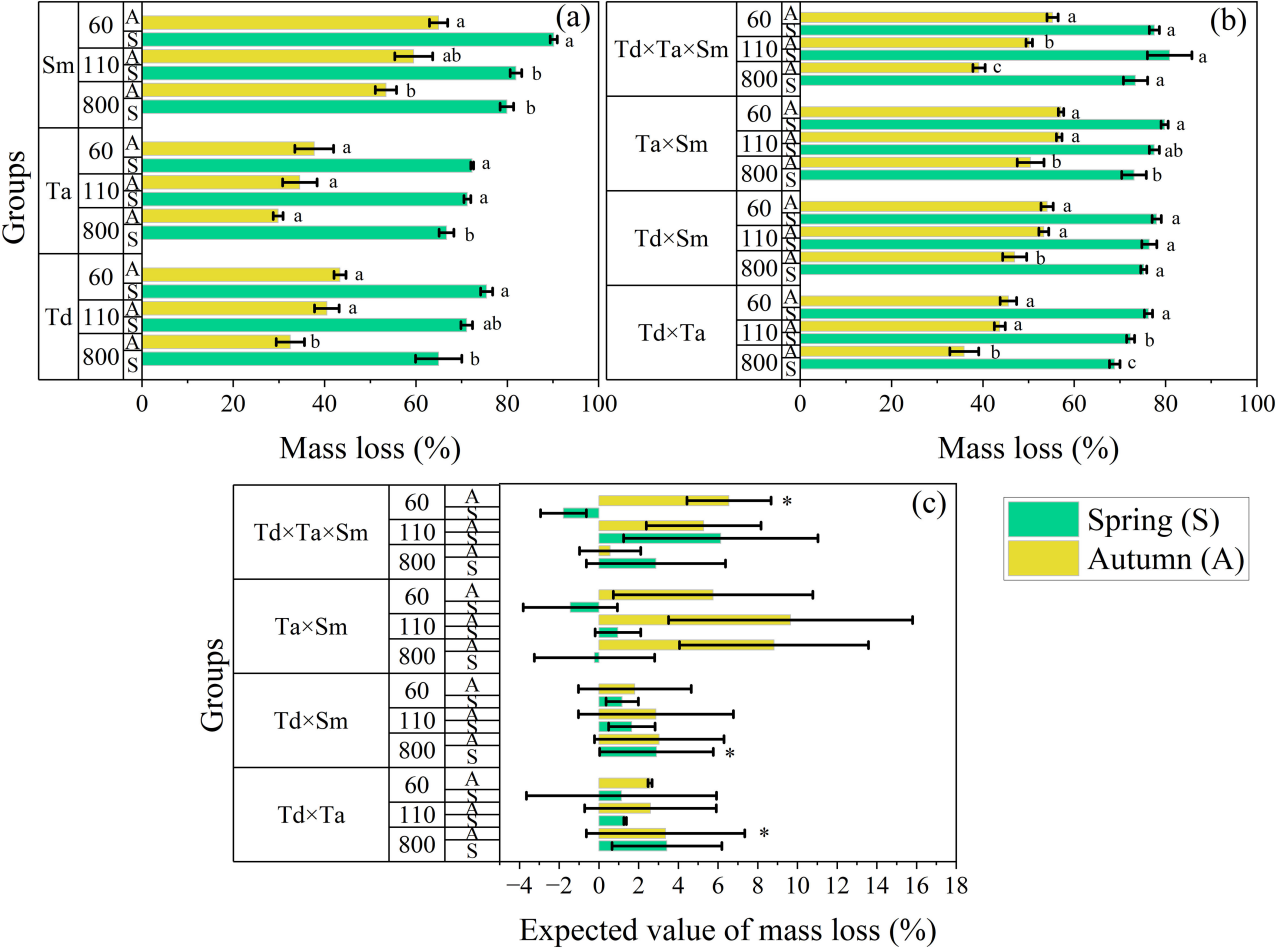
Grouped bars of mass loss of single litter treatments **(a)**, mixed litter treatments **(b)**, and comparison between the observed and expected value of mass loss of mixed litter treatments **(c)** in river ecosystems. Means with different lowercase letters represent significant differences at P < 0.05 according to the two-way ANOVA **(a, b)**. According to the independent sample t-test, statistical differences are significant at p < 0.05* **(c)**. Positive values indicate an observed value that exceeds its expected value, and vice versa **(c)**. The vertical whisker represents the standard error (black color). Abbreviations in the figure are *Taxodium distichum* (Td), *Taxodium ascendens* (Ta), and *Salix matsudana* (Sm). The Y-axis displays three elevations: 800, 110, and 60 m. Sample size was n = 336.

The mass loss of single species was the largest (90.19 ± 0.77%) in *S. matsudana* during spring at 60 m elevation and the lowest (29.82 ± 10%) in *T. ascendens* during autumn at 800 m elevation (**Figure 2**). However, this pattern was inconsistent across different seasons and elevations. The mass loss ratios were substantially higher in spring compared to autumn. Moreover, the mass loss of mixed species was the largest (80.89 ± 4.88%) in *T. distichum × T. ascendens × S. matsudana* during spring at 110 m elevation and the lowest (35.91 ± 3.18%) in *T. distichum × T. ascendens* during autumn at 800 m elevation (**Figure 2**). This pattern followed a trend: *T. distichum × T. ascendens × S. matsudana < T. ascendens × S. matsudana < T. distichum × S. matsudana < T. distichum × T. ascendens* at different seasons and elevations. Furthermore, this pattern followed the distinctive trend of 60 m < 110 m < 800 m elevations. The mass loss was substantially higher in spring compared to autumn. Comparing the observed and expected mass loss of mixed species showed that non-additive effects of mass loss were more obvious in *T. ascendens × S. matsudana* during autumn at 110 m (9.65 ± 6.14%) and 800 m (8.81 ± 4.75%) elevations. These non-additive effects became negative in certain observations: *T. ascendens × S. matsudana* at 800 m (-0.22 ± 3.02%) and 60 m (-1.44 ± 2.37%) elevations, and *T. distichum × T. ascendens × S. matsudana* at 60 m elevation (-1.78 ± 1.15%) during spring. Additionally, standard error ranges were notable in *T. ascendens × S. matsudana* arrangements. However, the limited arrangements showed statistical differences, and most showed insignificant differences (**Figure 2**).

**Figure 3:**
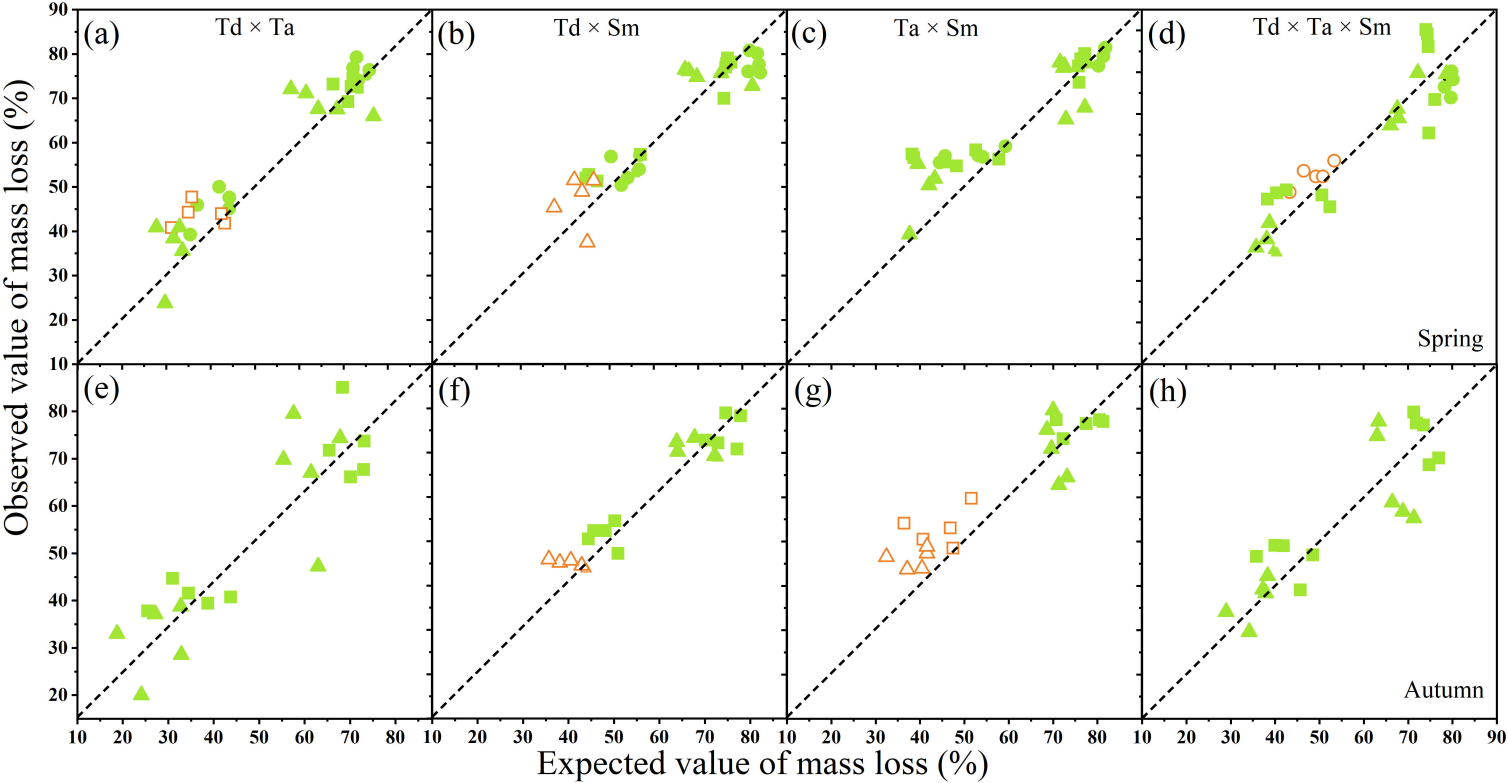
The non-additive effects of mixed litter treatments on the mass loss of leaf species in spring **(a-d)** and autumn **(e-h)** in river ecosystems. Abbreviations used are *Taxodium distichum* (Td), *Taxodium ascendens* (Ta), and *Salix matsudana* (Sm). The abscissa represents the expected mass loss of mixed samples, and the ordinate is their measured mass loss. A circle represents 60 m elevation, a square represents 110 m elevation, and a triangle represents 800 m elevation. The hollow shape of a circle, triangle, or square indicates a significant non-addition. Sample size was n = 192.

The comparison of the non-additive effects on mass loss of mixed litter treatments of pine and broadleaf species for spring and autumn is shown in **Figure 3**. The coniferous mixture *T. distichum* × *T. ascendens* showed synergistic effects across all sampling seasons and elevations. The results indicated that the mixed *T. distichum* × *T. ascendens* of two conifer species promoted the decomposition of single leaf *T. distichum* and *T. ascendens*. The coniferous and broad-leaved mixture *T. distichum* × *S. matsudana* showed non-additive effects across all sampling seasons and elevations. Similarly, the coniferous and broad-leaved mixture *T. ascendens* × *S. matsudana* promoted the decomposition of the single leaf of *T. ascendens*, influencing the mass loss with sampling season and elevation. The antagonism of the three mixed *T. distichum* × *T. ascendens* × *S. matsudana* occurred, while a synergistic effect occurred in the decomposition across all sampling seasons and elevations.

### 3.3 Nutrient release of single and mixed litter treatments at different elevations under seasonal changes

**Figure 4:**
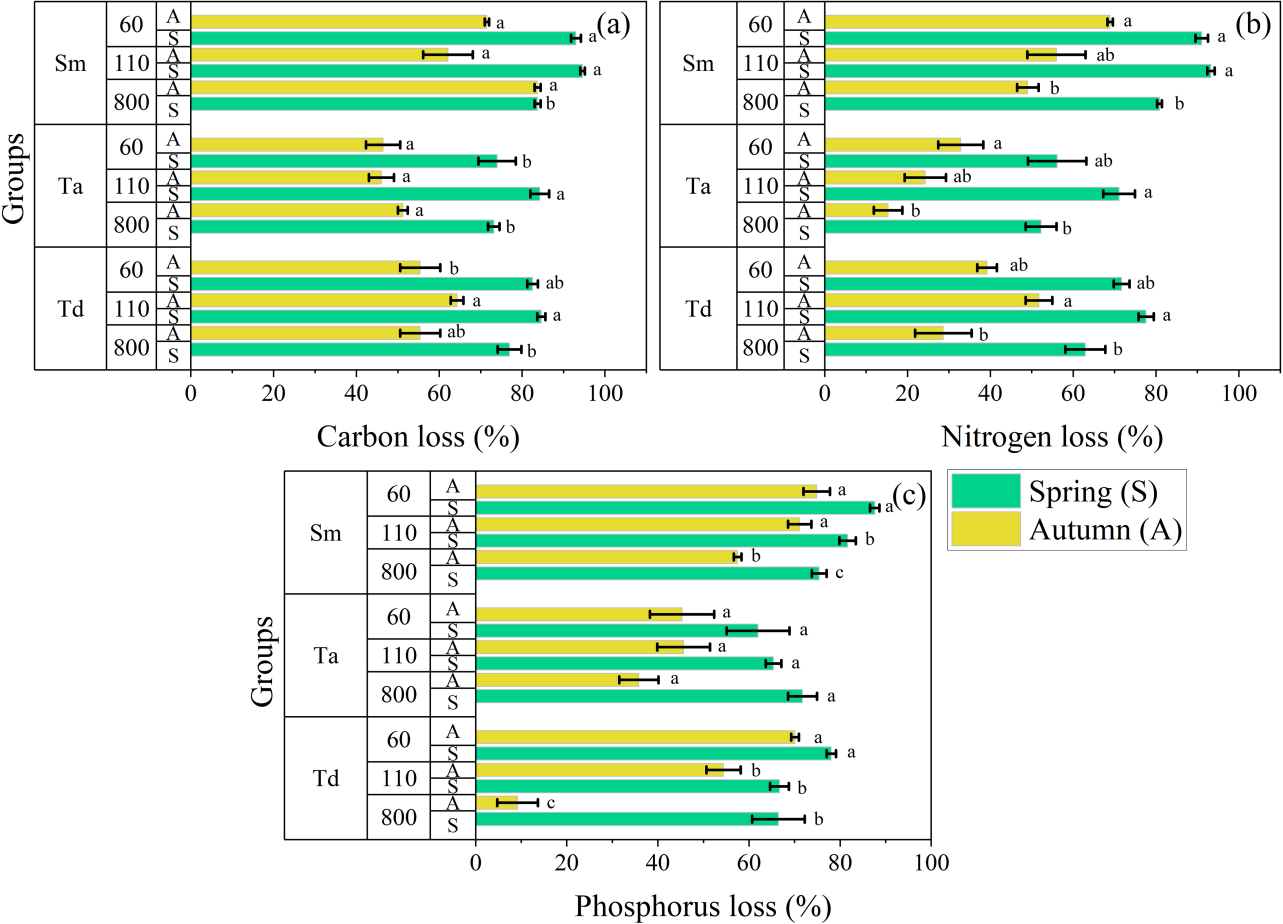
The bar graph groups the rates of element concentration loss for carbon **(a)**, nitrogen **(b)**, and phosphorus **(c)** in single litter treatment in river ecosystems. Means with different lowercase letters indicate significant differences at p < 0.05, according to the two-way ANOVA; abbreviations include *Taxodium distichum* (Td), *Taxodium ascendens* (Ta), and *Salix matsudana* (Sm). The vertical whisker represents the standard error (black color). The Y axis displays three elevations: 800, 110, and 60 m, respectively. Sample size was n = 144.

All foliar types significantly affect the release of C, N, and P, while different litter treatments, elevations, and their interactions significantly affect their release rates (**Figure 4**). The C release rate of single species was the largest (94.60 ± 0.58%) in *S. matsudana* during spring at 110 m elevation and the lowest (46.01 ± 3.02%) in *T. ascendens* during autumn at 110 m elevation (**Figure 4**). The pattern of C release followed the same pattern across *S. matsudana*, *T. ascendens*, and *T. distichum*: 110 m < 60 m < 800 m. However, this pattern was inconsistent across different seasons and elevations. The C release rate was substantially higher in spring compared to autumn. The N release rate of single species followed the same pattern as C and was the largest (93.27 ± 0.86%) in *S. matsudana* during spring at 110 m elevation. It was lowest (15.30 ± 3.44%) in the same *T. ascendens* during autumn at 800 m elevation (**Figure 4**). The pattern of N release followed the same patterns across *S. matsudana*, *T. ascendens*, and *T. distichum*: 110 m < 60 m < 800 m. The N release rate was considerably higher in spring compared to autumn. The P release rate of single species followed the same pattern as that of C and N. It was the largest (87.62 ± 1.02%) in *S. matsudana* during spring at 60 m elevation. However, it was lowest (9.20 ± 4.47%) in *T. distichum* but not in *T. ascendens* during autumn at 800 m elevation (**Figure 4**). The pattern of P release followed the patterns of *S. matsudana* and *T. distichum*: 60 m < 110 m < 800 m, and this pattern was reversed for *T. ascendens*. Importantly, the P release rate was considerably higher in spring compared to autumn.

**Figure 5:**
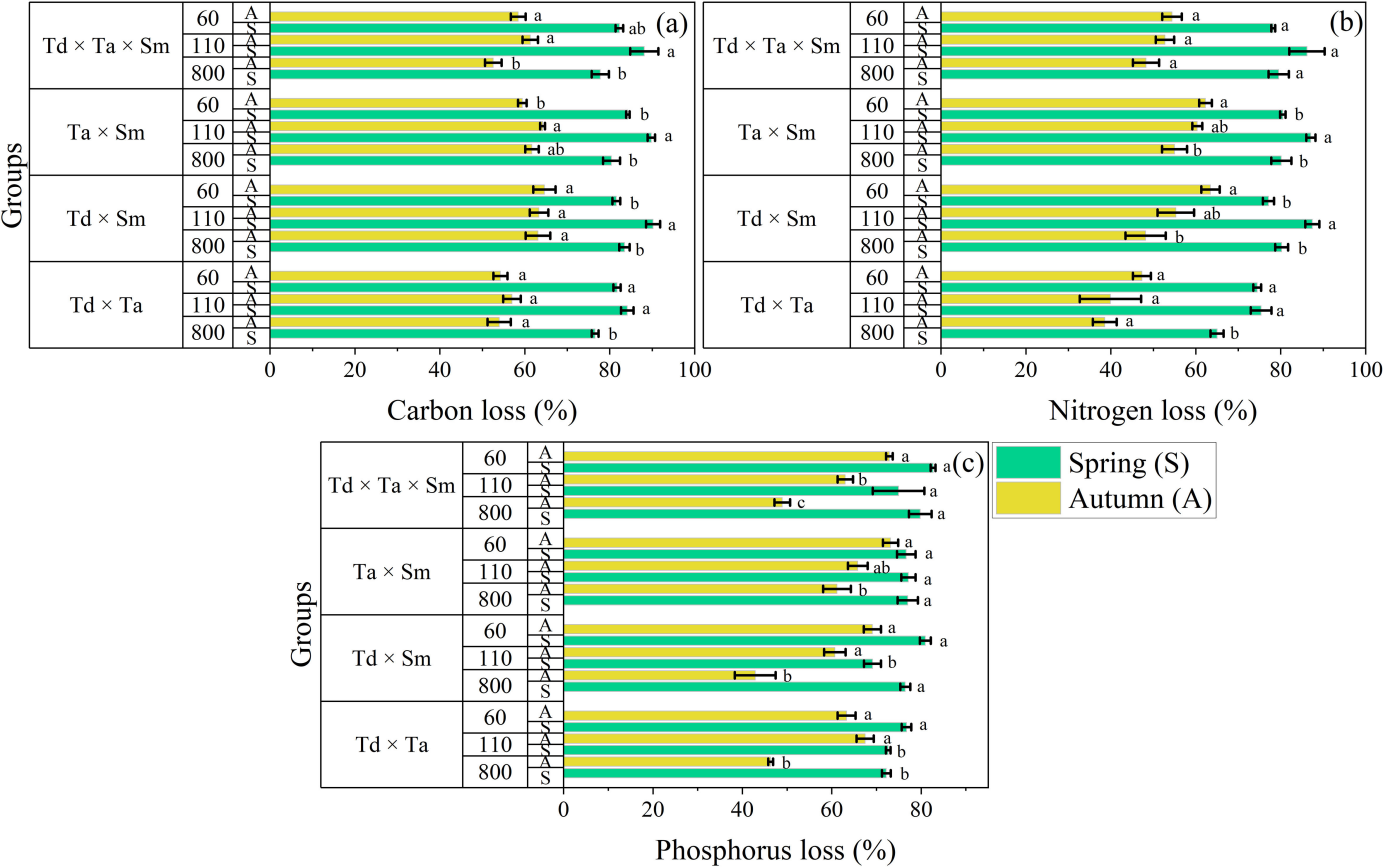
The bar graph groups the rates of element concentration loss for carbon **(a)**, nitrogen **(b)**, and phosphorus **(c)** in mixed litter treatment in river ecosystems. Means with different lowercase letters indicate significant differences at p < 0.05, according to the two-way ANOVA; abbreviations include *Taxodium distichum* (Td), *Taxodium ascendens* (Ta), and *Salix matsudana* (Sm). The vertical whisker represents the standard error (black color). The Y axis displays three elevations: 800, 110, and 60 m, respectively. Sample size was n = 192.

The C release rate of mixed species was the largest (90.20 ± 1.65%) in *T. distichum* × *S. matsudana* during spring at 110 m elevation and the lowest (52.56 ± 1.95%) in *T. distichum* × *T. ascendens* × *S. matsudana* during autumn at 800 m elevation (**Figure 5**). The pattern of C release followed the same pattern across all mixture combinations: 110 m < 60 m < 800 m. However, this pattern was inconsistent across different seasons and elevations. The C release rate was significantly higher in spring compared to autumn. The N release rate of mixed species followed the same pattern as C and was the largest (87.42 ± 1.64%) in *T. distichum* × *S. matsudana* during spring at 110 m elevation. It was lowest (38.56 ± 2.82%) in *T. distichum* × *T. ascendens* during autumn at 800 m elevation (**Figure 5**). The pattern of N release followed dissimilar patterns across mixture combinations: 110 m < 800 m < 60 m. The N release rate was considerably higher in spring than autumn. The P release rate of mixed species showed a distinctive pattern different from C and N. It was the largest (82.67 ± 0.55%) in *T. distichum* × *T. ascendens* × *S. matsudana* during spring at 60 m elevation and the lowest (42.87 ± 4.46%) in *T. distichum* × *S. matsudana* during autumn at 800 m elevation (**Figure 5**). A dissimilar pattern was observed for P release, with 110 m < 60 m < 800 m, differing for *T. distichum* × *S. matsudana*. Importantly, the P release rate was substantially higher in spring compared to autumn.

### 3.4 Effects of different treatment of leaf mixing on nutrient release at different elevations under seasonal changes

**Figure 6:**
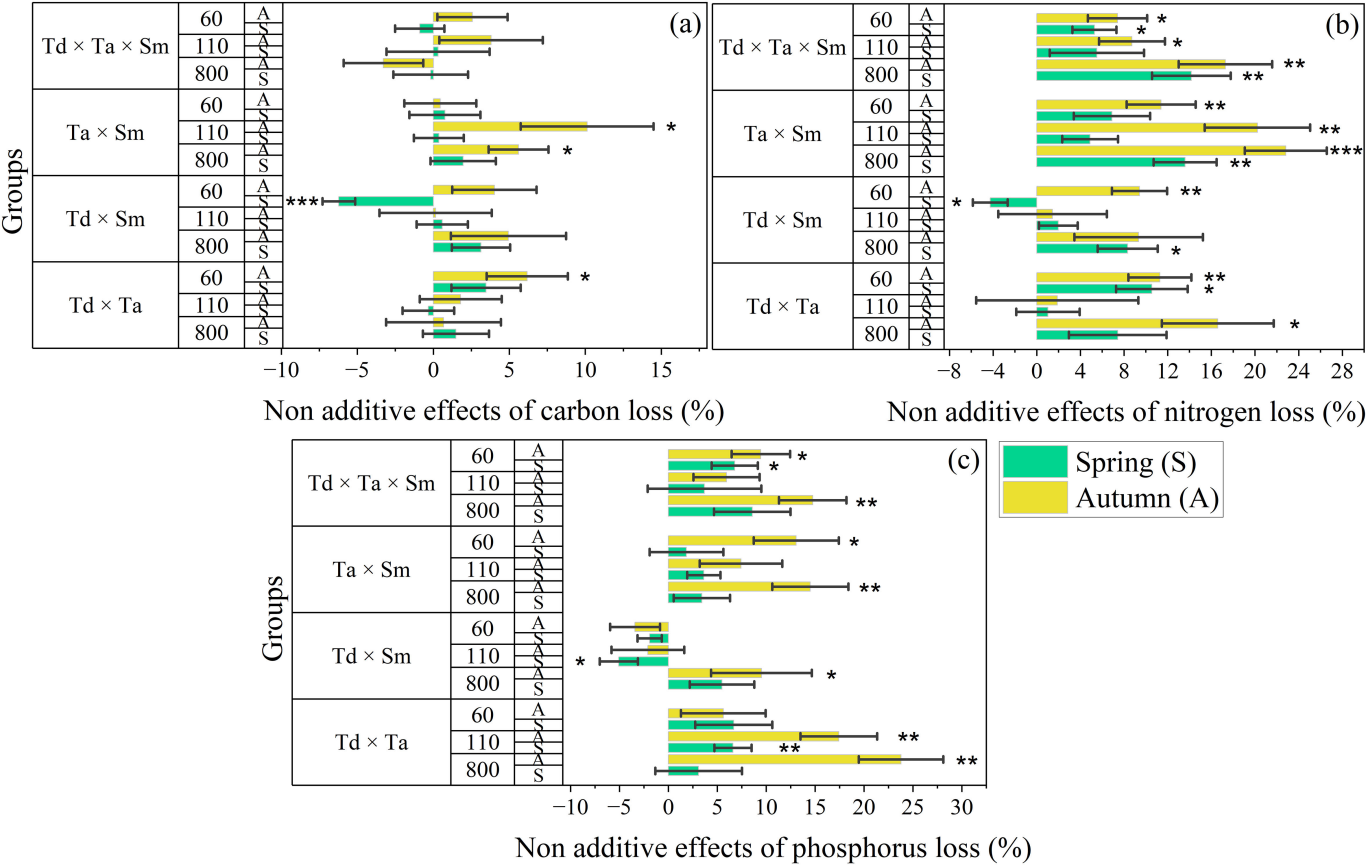
The bar graph groups the non-additive effects of carbon **(a)**, nitrogen **(b)**, and phosphorus **(c)** on mixed litter treatment in river ecosystems. According to the independent sample t-test, statistical differences are significant at p < 0.05*, p < 0.01**, and p < 0.001***. Abbreviations in the figure are *Taxodium distichum* (Td), *Taxodium ascendens* (Ta), and *Salix matsudana* (Sm). The vertical whisker represents the standard error (black color). The Y-axis displays three elevations: 800, 110, and 60 m. Sample size was n = 192.

The non-additive effects of C, N, and P release from all litter treatments at different elevations and seasons are shown in **Figure 6**. The C release rate of each mixed type of elevation and season has a non-additive effect, but the effect varies with sampling season and elevation (**Figure 6**). The non-additive effects of C release in mixed species were the largest (10.12 ± 4.37%) in *T. ascendens × S. matsudana* during autumn at 110 m elevation and the lowest (-6.22 ± 1.07%) in *T. distichum* × *S. matsudana* during spring at 60 m elevation (**Figure 6**). The pattern of non-additive effects of C release followed dissimilar patterns across all mixture combinations and mostly had positive values. However, this pattern also had some negative values, mostly from *T. distichum × T. ascendens* × *S. matsudana*. Moreover, limited combinations showed statistical differences at p<0.05. The non-additive effects of N release in mixed species followed a different pattern from C and was the largest (22.81 ± 3.74%) in *T. ascendens* × *S. matsudana* during autumn at 800 m elevation. However, it was lowest (-4.26 ± 1.49%) in *T. distichum* × *S. matsudana* during spring at 60 m elevation (**Figure 6**). The pattern of non-additive effects of N release followed distinctive patterns across mixture combinations: *T. ascendens* × *S. matsudana* < *T. distichum* × *T. ascendens* × *S. matsudana < T. distichum* × *T. ascendens* < *T. distichum* × *S. matsudana*. This pattern mostly had positive values, and most combinations showed statistical differences at p<0.05. The non-additive effects of P release in mixed species followed a distinctive pattern different from C and N. It was the largest (23.79 ± 4.31%) in *T. distichum* × *T. ascendens* during autumn at 800 m elevation and the lowest (-5.06 ± 1.94%) in *T. distichum × S. matsudana* during spring at 110 m elevation (**Figure 6**). The pattern of non-additive effects of P release followed dissimilar patterns, mostly positive except for *T. distichum* × *S. matsudana*, which were overwhelmingly negative. Most combinations showed statistical differences at p<0.05.

## 4 Discussion

This study investigates the influence of elevation, season, and litter treatments on litter decomposition and associated nutrient fluxes. In aquatic systems, litter decomposition is closely linked to both physical and chemical properties (**Tables 1**–**4**), as well as environmental factors such as elevation, temperature, humidity, and water characteristics—including pH, dissolved oxygen, water temperature, and flow rate (Chen et al., 2024a; Zheng et al., 2024). Biological factors, such as benthic invertebrates and microorganisms, also play a crucial role (Li et al., 2024a; Putra et al., 2023). Physical properties of leaves—like texture, thickness, hardness, wax presence, and cuticle thickness—significantly influence decomposition rates (Rahman et al., 2023). Research indicates that leaves with a softer texture and lower leathery degree decompose more rapidly than those with tougher textures and greater rigidity (Grootemaat et al., 2015; Hoeber et al., 2020), regardless of whether they are in flowing or still-water environments. Leaves with lower toughness break down faster. In our study, *S. matsudana*, which has softer and thinner leaves, exhibited higher decomposition rates than T. distichum and *T. ascendens*. The thicker cuticles of the latter species hinder fungal invasion and water absorption, slowing decomposition. This pattern was consistent across all sampling stages and elevations (**Figure 2**), likely due to differences in leaf tissue structure and resistance to physical fragmentation (Salgado-Luarte et al., 2023).

The chemical composition of leaves also plays a crucial role in decomposition rates, with factors such as N, P, lignin, cellulose, and the C/N ratio being key determinants (He et al., 2019; Talbot and Treseder, 2012). High lignin or tannin content generally slows down decomposition, whereas a low C/N ratio accelerates the process (Bertrand et al., 2006). In our study, *S. matsudana*, which had a higher N concentration and lower C/N ratio, exhibited significantly greater dry mass loss than T. distichum and *T. ascendens*, both with lower N concentrations. Higher N availability promotes microbial growth on leaf surfaces, leading to increased microbial reproduction and, consequently, faster decomposition (Wu et al., 2023). Previous research on mixed-leaf decomposition in forest ecosystems suggests that approximately 67% of mixed litter exhibit diverse effects during decomposition (Chapman and Koch, 2007; Zhang et al., 2017). This occurs because high-quality leaves (those with higher nutrient content) transfer nutrients to lower-quality leaves, enhancing their nutritional availability and mitigating nutrient limitations for microorganisms. Specifically, about 64.3% of coniferous and broadleaf mixtures accelerate decomposition, highlighting their overall positive effect (Qiu et al., 2023). Our findings, spanning different elevations and seasons, aligning with those observed in KRSs, demonstrating non-additive effects in mixed decomposition across all sampling periods, though the magnitude of these effects varies (**Figure 3**).

Among the litter treatments studied, *T. distichum* and *T. ascendens* have smaller, thicker cuticles and more rigid textures, which may slow the leaching rate during the initial stage of decomposition (Chen et al., 2024a). In contrast, mixed-leaf treatments, such as coniferous-broadleaf mixtures (*T. distichum × S. matsudana and T. ascendens × S. matsudana*), exhibited higher N concentrations and lower C/N ratios compared to coniferous mixtures (*T. distichum × T. ascendens*). High-quality litter, characterized by elevated N concentrations, attracts more decomposers, who may transfer nutrients to adjacent leaves through leaching or mycelial networks, accelerating decomposition (Lyu et al., 2019). Our results indicate that dry mass loss in coniferous-broadleaf mixtures was consistently greater than in coniferous mixtures across different seasons and elevations (**Figure 4**). This suggests that similar processes observed in forest ecosystems also occur in aquatic environments but with faster leaching and higher decomposition rates in flowing-water zones. Environmental factors and decomposition cycles further influence leaf decomposition (Putra et al., 2023). In KRSs, leaves at lower elevations experience prolonged flooding periods, which enhance decomposition. For example, during summer, the mass loss of all leaf types at 60 m was significantly higher than at other elevations. This confirms that extended flooding accelerates decomposition. Additionally, stable water temperatures, continuous water supply, and rapid nutrient cycling in aquatic environments support this process. Flowing water enhances decomposer activity, promoting bacterial and fungal colonization (Li et al., 2024a). Temperature also plays a crucial role, as higher water temperatures stimulate microbial activity, further accelerating decomposition (Calero Preciado et al., 2021).

The dynamics of element release during leaf decomposition in KRSs are influenced by both leaf characteristics and aquatic environmental factors (Zhang et al., 2017). Our results revealed distinct patterns of element release across different seasons and elevations, with notable species-specific variations (**Figure 6**). Decomposition-driven release of C, N, and P plays a crucial role in ecosystem biogeochemical cycles. Changes in hydrological patterns within KRSs directly impact leaf decomposition, influencing material circulation and interactions between terrestrial and aquatic ecosystems (Chen and Arif, 2025; Ford et al., 2019). Nutrient fluxes generally increase over time, particularly at lower elevations during the summer, driven by leaf matrix quality, environmental conditions, and the specific elements involved. Biodiversity plays a key role in nutrient dynamics during mixed-leaf decomposition, as species interactions create mixing effects. High-nutrient-content leaves supply nutrients to lower-nutrient-content leaves, slowing nutrient release and mitigating microbial nutrient limitations (Du et al., 2020). This nutrient redistribution facilitates decomposition and enhances nutrient release. Our study found that nutrient flux release rates were higher in coniferous-broadleaf mixtures than in coniferous mixtures, suggesting that mixed-leaf treatments create a more favorable environment for decomposers. This accelerated both decomposition and nutrient release, highlighting the ecological significance of species diversity in nutrient cycling.

Spring and summer rainstorms, which lead to increased discharge, are closely associated with rapid increases in the nutrient flux released from leaves (Oehler et al., 2018; Zhao et al., 2021). Summer conditions promote decomposition faster than autumn. As autumn temperatures drop and precipitation increases at higher elevations, leaf nutrient flux declines. Furthermore, significant rainfall events marking the end of the dry period can elevate surface water nutrient concentrations (Fong et al., 2020). In contrast, autumn brings only minor increases in river water nutrient concentrations within KRSs. Our measurements confirm previous studies indicate that large discharge events caused by heavy summer precipitation quickly flush away nutrients from surface soils, resulting in rapid nutrient export (Skidmore et al., 2023). Elevated discharge is linked to higher nutrient concentrations. This suggests that these short-lived flow events account for significant portions of the annual nutrient flux in KRSs. This finding aligns with other river studies (Oehler et al., 2018). Nutrient flux concentrations vary with elevation, and organic matter composition changes based on litter treatment and season. Lower nutrient flux was observed during the autumn season, while higher values occurred during the spring season at lower elevations. During spring, surface soils and vegetation contribute substantially to higher nutrient flux values. Numerous watershed studies have established that spring typically hosts an abundance of nutrient sources from surface soils and vegetation (Li et al., 2021; Ni and Li, 2020; Yi et al., 2021). This phenomenon contributes to greater nutrient flux than at any other time of year. Thus, we conclude that during the high-flow spring—and during some large summer rainstorms with rapid, shallow flows—KRSs along the Li River experience increased nutrient flux from surface soil and plant litter, which tends to be high in C but low in N. In contrast, *in-situ* production during low-flow conditions in autumn significantly reduces flux ratios.

Future research could focus on the long-term implications of climate change on KRSs, particularly the effects of changes in temperature and precipitation (Lian et al., 2020). Additionally, it is crucial to assess the impact of vegetation and land use changes on biogeochemical outcomes within KRSs (Chi and He, 2023). Added field data to hydrological and ecological models could help make better predictions about biogeochemical dynamics in KRSs, which would lead to better conservation and management plans (Johnson et al., 2021).

## 5 Conclusion

This study highlights the critical roles elevation and seasonality play in shaping leaf decomposition and nutrient release within the Li River karst systems. The results revealed significant interactions between litter treatments, elevation, and season. These interactions were notably higher in spring than in autumn. At lower elevations, decomposition and nutrient release were more pronounced, highlighting the significant influence of elevation on temperature and moisture variations. Distinct patterns emerged among litter treatments, with species like *Salix matsudana* exhibiting the highest decomposition rates at lower elevations in spring. In contrast, *Taxodium ascendens* showed the lowest rates at higher elevations in autumn. Mixed-leaf treatments displayed both synergistic and antagonistic effects, depending on species and environmental context. Importantly, non-additive effects on nutrient release and mass loss were observed in mixed-species treatments, reflecting complex interspecies interactions that fluctuate with elevation and season. These findings provide valuable insights into ecological restoration strategies in karst systems, advocating for approaches that consider both spatial and temporal dynamics. Effective management must integrate knowledge of how elevation and seasonality influence decomposition to preserve and enhance ecosystem health. Future research can explore the long-term impacts of these patterns and their interactions with broader climatic and anthropogenic changes, aiming to develop sustainable conservation and restoration practices for karst river systems.

## Data Availability

The raw data supporting the conclusions of this article will be made available by the authors, without undue reservation.
