# Supplementary material for: Elevation and seasonality modulate the leaf decomposition rates and nutrient flux of diverse species and species richness in karst river systems across China
Source: Front Plant Sci. 2025 Jun 4;16:1543011. doi: 10.3389/fpls.2025.1543011 (PMC12174984; doi:10.3389/fpls.2025.1543011)
Supplement: Supplementary file 1 [file Table1.docx]

Supplementary Material

# Supplementary Figure


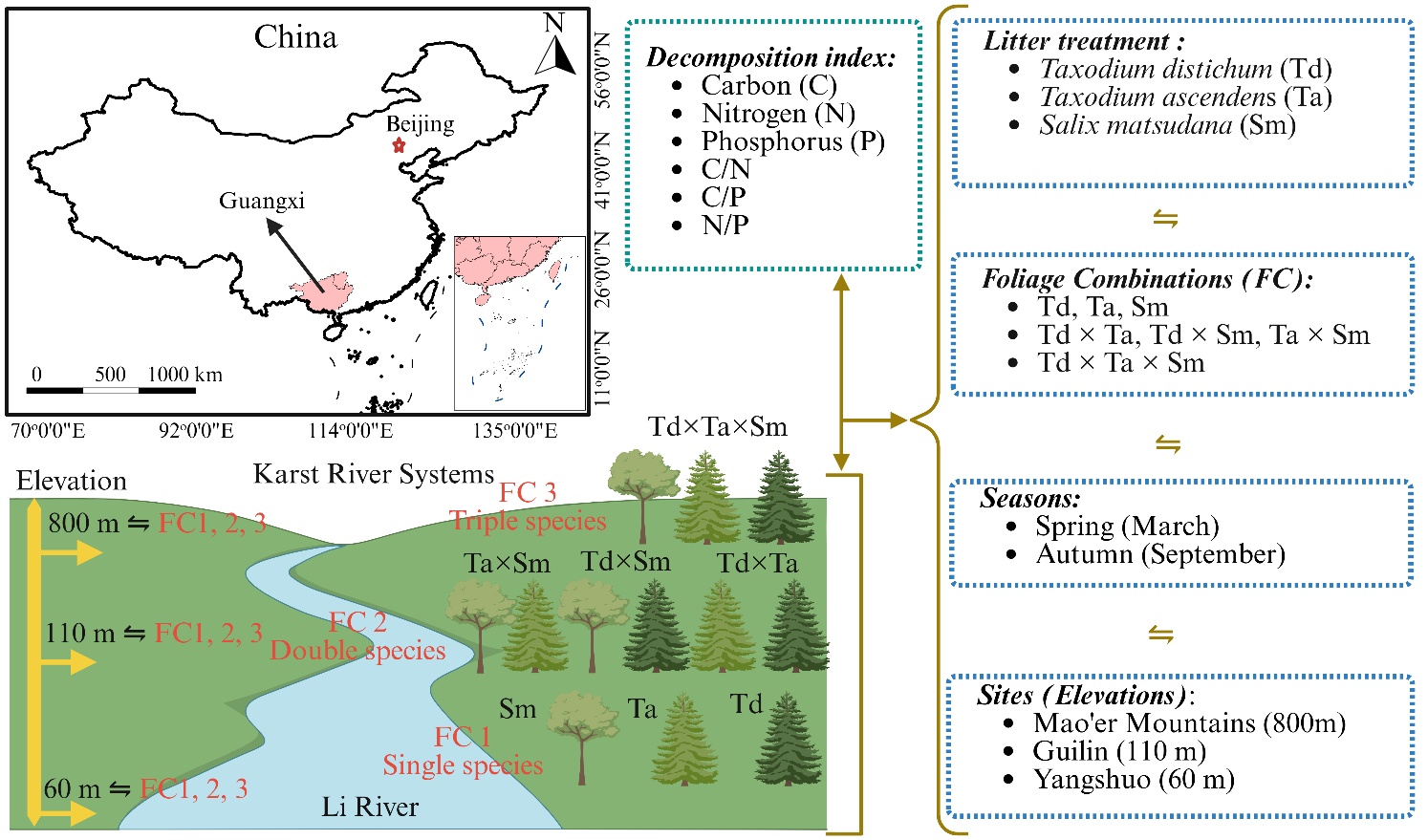


**Figure S1.** The location of the study area and experimental design across elevation gradients in the Li River karst system.

# Supplementary Table

**Table S1.** Water characteristics in the Li River Basin during decomposition in river ecosystems.

| Elevation  (m) | Temperature (℃) | | | Dissolved Oxygen (mg·L^-1^) | | | Electric Conductivity (μS·m^-1^) | | |
| --- | --- | --- | --- | --- | --- | --- | --- | --- | --- |
|  | Average | Maximum | Minimum | Average | Maximum | Minimum | Average | Maximum | Minimum |
| 800 | 15.8 | 32.2 | 0.0 | 8.6 | 11.3 | 6.5 | 101.6 | 150.8 | 80.3 |
| 110 | 21.7 | 35.8 | 4.3 | 8.1 | 10.5 | 6.0 | 256.6 | 330.4 | 210.5 |
| 60 | 22.4 | 37.5 | 2.1 | 8.1 | 10.7 | 6.0 | 226.6 | 300.6 | 190.2 |
